# Community engagement and feedback of results in the H3Africa AWI-Gen project: Experiences from the Navrongo Demographic and Health Surveillance site in Northern Ghana

**DOI:** 10.12688/aasopenres.13081.1

**Published:** 2021-03-19

**Authors:** Godfred Agongo, Cornelius Debpuur, Lucas Amenga-Etego, Engelbert A. Nonterah, Michael B. Kaburise, Abraham Oduro, Michele Ramsay, Paulina Tindana

**Affiliations:** 1 Navrongo Health Research Centre, Navrongo, Ghana; 2 West African Centre for Cell Biology and Infectious Pathogens, Department of Biochemistry, University of Ghana, Accra, Ghana; 3 Julius Global Health, Julius Center for Health Sciences and Primary Care, University Medical Center Utrecht, Utrecht University, Utrecht, The Netherlands; 4 Sydney Brenner Institute for Molecular Bioscience, Faculty of Health Sciences, University of Witswatersrand, Johannesburg, South Africa; 5 Health Policy, Plannng and Management, School of Public Health, College of Health Sciences, University of Ghana, Legon/Accra, Ghana

**Keywords:** Community engagement, feedback of results, Ghana, genomics

## Abstract

Community and Public engagement (CE) have gained traction as an ethical best practice for the conduct of genomics research, particularly in the context of Africa. In the past 10 years, there has been growing scholarship on the value and practice of engaging key stakeholders including communities involved in genomics research. However, not much has been documented on how research teams, particularly in international collaborative research projects, are navigating the complex process of engagement including the return of key research findings. This paper is part of a series of papers describing the CE processes used in the AWI-Gen study sites. We describe the key processes of engagement, challenges encountered and the major lessons learned. We pay particular attention to the experiences in returning research results to participants and communities within the Demographic and Health Surveillance site in northern Ghana.

## Disclaimer

The views expressed in this article are those of the author(s). Publication in AAS Open Research does not imply endorsement by the AAS.

## Introduction

The AWI-Gen Study is a collaboration between the University of the Witwatersrand (Wits) and the International Network for the Demographic Evaluation of Populations and Their Health in Low- and Middle-Income Countries (INDEPTH) ( Ramsay, 2015; Ramsay *et al.,* 2016). It is part of the Human Heredity and Health in Africa (H3Africa) consortium which is funded by the Wellcome Trust (United Kingdom) and the National Institutes of Health (NIH) (United States) ( H3Africa Consortium *et al.,* 2014; H3Africa, 2011). The AWI-Gen study capitalizes on the unique strengths of existing longitudinal cohorts, including the urban Soweto birth cohort study site in South Africa and INDEPTH demographic and health surveillance member centers in Nairobi, Kenya, Navrongo, Ghana, Nanoro, Burkina Faso, and rural Agincourt and Dikgale in South Africa. These centers offer established infrastructure, trained fieldworkers, long-standing community engagement strategies, as well as detailed longitudinal phenotypic data, focusing on obesity and cardiometabolic health ( Ramsay *et al.,* 2016).

The first phase of the AWI-Gen study was conducted between 2014 and 2017 and was divided into two arms: the population structure arm and the cardiometabolic disease and body composition arm. The population structure arm involved random sampling of adults 18 years and above; 30 trios and 40 unrelated individuals who were recruited from the two Kassena-Nankana districts (KNDs). In the second arm of the study a population-based cross-sectional study of older adults was carried out in the same two KNDs. Multi-stage random sampling was done and 2200 participants (roughly equal male and female participants) were selected. Eligible individuals were residents who have lived within the study area for at least 10 years. At the completion of the first phase of the study in 2017, 2016 participants were recruited into this arm of the study.

The study was approved by the Navrongo Health Research Centre Institutional Review Board (ID No: NHRCIRB178), the Ghana Health Service Ethics Review Committee (ID No: GHS-ERC:05/05/2014) and the Human Research Ethics Committee (HREC) of the University of the Witwatersrand (ID No: M12109, renewal M170880). In addition to these approvals, the study team also consulted with district health authorities, particularly the management team of the district hospital, regarding implementation of the study. This was important as the AWI-Gen study involved older adults (age 40–60 years) and the recruitment center was located within the premises of the hospital.

Similar to the other AWI-Gen collaborative research centers, community engagement was a key component of the implementation of the study in the Kassena-Nankana population. Prior to and during recruitment of study participants, various activities were undertaken to promote community understanding, acceptance and participation in the study to support the successful implementation of the study. These activities collectively constituted the Community Engagement model of the AWI-Gen study in Navrongo. In this paper, we describe our community research experiences and the four steps CE process we used for the AWI-Gen study (1. Meeting with chiefs and elders; 2. Community durbars; 3. Compound and household visits; and 4. Group information sessions and individual informed consent), as well as our experiences with returning research results to participants and communities. We highlight some of the challenges and key lessons learned.

## Community research experiences in the Kassena-Nankana districts

The Kassena-Nankana districts, which form the Navrongo Health and Demographic surveillance system (NHDSS) area, are located in the Upper East region of northern Ghana and share borders with neighboring Burkina Faso to the North. With a current population of 165,000, it is described as mainly rural with two main ethno-linguistic groups, the Kassenas and the Nankani, and the minority Buli speaking group. Communities in these districts are familiar with the conduct of health research activities mainly through the work of the Navrongo Health Research Centre (NHRC) which has been in existence since 1989. The NHRC started as a field research site for a Vitamin A supplementation trial and has evolved over the years to become a reputable research centre of the Ghana Health Service involved in several epidemiological studies, clinical trials and social science studies ( Oduro *et al.,* 2012). Some of the genomic studies that have been conducted within this district include the MalariaGen project ( MalariaGEN, 2008), a case-control study which involved children under five. The AWI-Gen study was the first genomics study to involve adults between the ages of 40–60 years ( Ramsay *et al.,* 2016). While this rich research infrastructure and community experience provided a good environment that facilitated the conduct of the AWI-Gen study, the CE processes also highlighted some persisting ethical issues around consent, community engagement and feedback of research results.

## The AWI-Gen Community engagement process

In implementing the AWI-Gen study, we adopted the CE practices routinely used for studies conducted through the NHRC including genomic studies such as the MalariaGen study conducted in 2010 ( Tindana *et al.,* 2012). This approach is closely aligned with the traditional authority structures as well as decision making practices in the community. This process has been used for previous studies and has been described elsewhere ( Tindana *et al.,* 2011). It begins with community entry where researchers seek the permission of chiefs and elders of all target communities culminating in a community durbar (see community durbar section below), meeting with identifiable community groups (e.g. women groups), compound and household meetings and individual consent. These processes are often facilitated by a team of researchers and community engagement and communication staff who are also natives and currently live in these communities and who speak the local languages. In what follows, we describe how the target community was defined, what methods were used for engagement and the challenges encountered.

### Defining the target community

Although there is no generally accepted definition of community, researchers in the Navrongo AWI-Gen study broadly defined community as residents of the Navrongo Health and Demographic surveillance catchment area. As the study was being implemented in the KNDs, such a definition largely aligned with the common definitions of community and included the elements of geographic location with shared culture and traditions, shared economy/resources and self-identification that are essential in most definitions of community ( Tindana *et al.*, 2011). The target population was then defined as all adult men and women between the ages of 40–60 years who reside in communities within the KNDs. The broad definition of community informed the community engagement processes used by the study team.

### The community entry process

Following the NHRC model of engagement, the first stage of the engagement process was to organise community entry meetings with each of the 10 paramount chiefdoms of the KNDs to explain the study to the paramount chiefs and elders, and to seek their permission to approach other members of the community. Community entry is the process of meeting with and seeking permission from community leaders prior to initiating any activity including research ( Tareen & Abu Omar, 1997) and has been highlighted as a key process to the success of most community-based research projects and interventions ( Nyonator *et al.,* 2005). Like many African communities, the chiefs and elders are important representatives of community interests and key gatekeepers. Engaging with them prior to approaching individuals therefore helps to allay suspicion, to nurture trust, and to establish the researchers’ credibility. Multiple consultations and discussions with chiefs and residents also helped to establish mutual trust between researchers and the community, which has been sustained over the years.

At each of the ten AWI-Gen community entry meetings, three members of the NHRC team took turns to address the community leaders in the local language. All of them are natives of the KNDs and have worked with the communities for over two decades as researchers. Going through the engagement process therefore came naturally to them and the community leaders also recognized the team as one of their own. A team member provided highlights of NHRC’s research in the district to date, emphasizing projects that have influenced health policy, such as vitamin A supplementation, use of bednets for malaria prevention, the community health and family planning project, and meningitis and rotavirus studies. The member then underlined the fact that most of the research conducted thus far has focused on the health of children, to the neglect of adult health issues and indicated the AWI-Gen study was aiming at addressing this gap by focusing on the health of adults. Another member of the study team then followed with a general explanation of the key emerging adult health issues such as hypertension, diabetes and stroke, and the need to understand why some people get these diseases while others do not. A broad overview of the AWI-Gen study was presented highlighting the multi-site nature of the study, the two arms, the target population, sampling procedure and sample size, as well as the study procedures for each arm of the study. The description of the study, purpose and procedures were similar to those described in the study information sheets for the individual consent process. The team concluded their presentation by seeking the permission and approval of the chiefs and elders to conduct the study in their communities. This was followed by an open forum for discussion and for the leaders to seek clarification and make contributions and suggestions. Some of the questions raised by the community leaders went beyond the scope of the AWI-Gen study. For example, some community leaders raised concerns about the use of pesticides for vegetable cultivation and the potential harmful effects of this practice on the health of farmers and consumers. They recommended that the study should liaise with relevant stakeholders to address these issues. The community members also expressed their support for the AWI-Gen project and said most of the studies conducted at the NHRC were targeted at children. The study team received permission from all ten paramount chiefs and their elders who also pledged to support the study team in the implementation of the study.

### Utilizing traditional methods of engagement: The community durbar

Following community entry and approval from the community leaders, community durbars which involved a gathering of chiefs, elders, opinion leaders and community members, were held to allow the research team to present the proposed study to the extended members of various communities within the district. “A durbar is a formal community-wide gathering that includes cultural activities such as drumming and dancing and provides an opportunity for information to be shared with a large number of people simultaneously” ( Tindana *et al.,* 2011). The public deliberations during the durbars provided an opportunity for community members to express their views and concern about the proposed study and to also ask questions about research and non-communicable diseases in general. The durbars also served to mobilize community support for the study. This type of dialogue offered opportunities for deliberation to shape researchers’ views about how to design and conduct their research. About twenty community durbars were held in the study area with attendance ranging from 50 to 100 people per durbar. In some cases, large communities were split into sections and separate durbars organized in order to target specific groups like women’s groups within the community. The durbars were the first public discussion of the study at the community level.

### Meetings at the level of households

The third stage of the engagement process involved visits to selected compounds by field supervisors to inform them of their selection, explain the study to them and invite them to a recruitment center on a scheduled date. These home visits provided yet another opportunity to explain the study to the members of the residential unit and clarify issues that may not have been well understood at the community meetings.

Eligible participants selected from the HDSS database were visited at home by a field supervisor. These follow up visits to houses of selected individuals were more focused. Here, the information centered on the aims and objectives of the study, why and how the person was selected, study procedures and what is expected of the participant, the voluntary nature of the study, issues of confidentiality and the right to withdraw participation. Those who agreed to participate in the study were asked to meet at a designated location within the community to be transported to the recruitment Centre.

The CE activities stretched over the period from September 2014 till October 2015 when recruitment was completed. At the end of the recruitment, 2016 adults aged 40 to 60 years were enrolled into the cardiometabolic arm of the study while 30 family trios and 40 unrelated adults were recruited into the population genome structure arm of the study.

### Group information-sharing sessions

On the day of recruitment, group information sessions were conducted for batches of individuals arriving for screening at the recruitment centre. Again, the aims and objectives of the study, the sampling procedure, study procedures and what is expected of the participant, the voluntary nature of the study, the right to confidentiality and the right to withdraw participation were explained to groups of participants. The study team demonstrated the sample collection process by using the tubes and also addressed persistent community concerns about the quantity of blood used for research purposes. This repeated information-sharing session was to ensure that participants who missed the community durbars and household meetings had the opportunity to discuss the research and seek clarification before the individual consent process. After this session those who agreed to participate in the study were invited into a private room and taken through the individual consent procedures.

### The approach to individual informed consent

Sampling of eligible participants took place following approval from all the relevant Ethics Review committees. The NHDSS database was used as a sampling frame and individuals aged 40–60 years, and spoke Kassem, Nankani and Buli, were identified. A harmonized informed consent process was developed for implementation across all the AWI-Gen research sites and adapted to the local context.

Individual informed consent, in the local language, specifically addressed consent for health-related studies, future pharmacogenetic studies, data sharing and biobanking. Individual participants gave their informed consent by signing or thumb printing on an informed consent form in the language they understood and in the presence of a credible witness. Due to the multi-layered CE approach, almost all the individuals who reported at the recruitment centre were taken through group sensitization and went on to give individual informed consent.

### Key challenges

The CE activities were not without challenges. The AWI-Gen study represented the first major study in the KNDs to recruit healthy adults into a study involving physical measurements and a questionnaire for phenotype assessment and health history of individuals, and sampling of blood and urine for laboratory assays and genotype assessments ( Ali *et al.,* 2018). More importantly the multi-site nature of the study and the biobanking and data sharing associated with the study were new concepts, which had to be explained to community members at the various stages of the CE process. Additionally, there were challenges in explaining genomic and genetic terms and concepts in Kassem and Nankani - the major local languages of the study community. Ensuring that research participants have an adequate understanding of a study’s objectives is a challenge in any setting ( Tindana *et al.,* 2011). In this case, the difficulty was compounded by the absence of western modern scientific concepts from the community’s general realm of experience.

The use of traditional Kassena and Nankani lines of communication as part of the CE process presented logistical and efficiency challenges. At the logistical level, following local protocols required planning, flexibility, and funding. For example, organizing large-scale meetings with paramount chiefs and communities (durbars) required scheduling the event ahead of time. Several visits were often made to the community before a successful durbar was organized. Some events had to be postponed at the last minute due to funerals, bad weather, or some other unanticipated events happening within the community. Large events, transportation, and general arrangements all came at a cost. For this reason, it is essential that research teams carefully plan and budget for CE activities when applying for funding and be prepared to show adequate flexibility throughout the study duration.

## Responding to community concerns: Feedback of research results

While much of the literature on community engagement within the context of genomics has focused on the methods used by research teams, the literature is limited on how engagement is done beyond data and sample collection. Following the completion of the first phase of the AWI-Gen study, we received some anecdotal reports that participants in the community were expecting feedback from the study. Subsequently, a qualitative study conducted in 2017 to explore key stakeholders’ view on broad consent in genomics research further highlighted community expectation for feedback of results from the AWI-Gen study ( Tindana *et al.,* 2020).

These community expectations for feedback of results were not unique to the study communities within the Kassena-Nankana districts but were a recurring theme across all the six collaborating centres of the AWI-Gen study. The study team discussed these community expectations extensively during team meetings and arrived at the conclusion that there was an ethical obligation to respond to these issues. Funding was made available for this purpose.

In the Ghana study site, two main sets of activities took place between May and July 2018 to support the feedback exercise. The first set of activities was community durbars which aimed at reporting the aggregate findings of the first phase of the project to the community and eliciting their input on the successes and challenges associated with their involvement in the project. The second set of activities was feedback of research findings to individual study participants.

## Community feedback durbars

**Figure 1.**
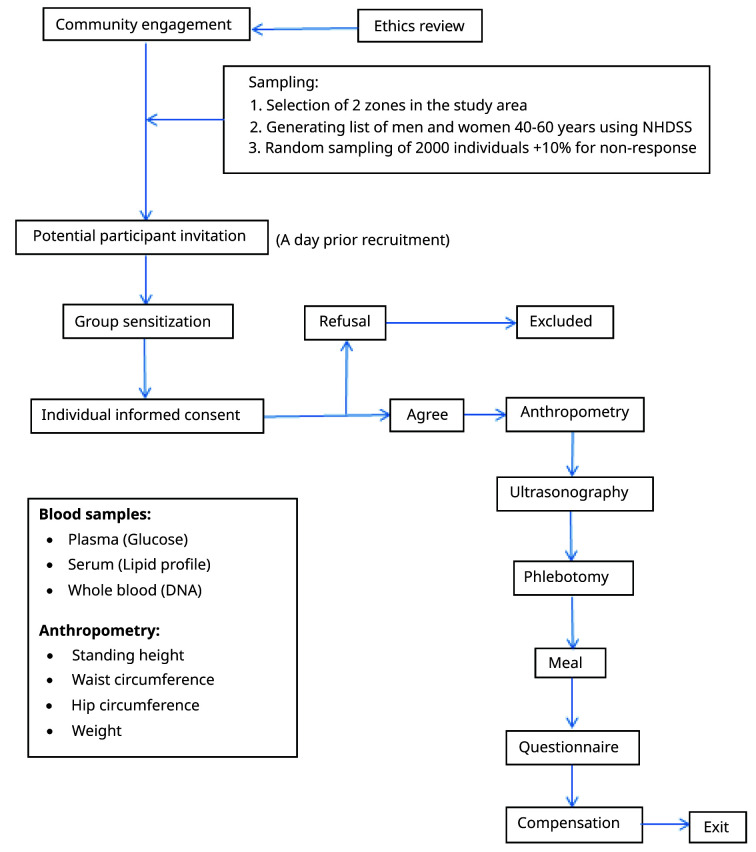
AWI-Gen community sensitzation and participant recruitment process. NHDSS: Navrongo Health and Demographic surveillance system.

Similar to the initial community sensitization and recruitment stage of the project ( Figure 1), community durbars were also organized at the feedback stage of the project. Overall, six durbars were organized strategically in the West, North, South and East zones of the NHDSS catchment area based on the distribution of the AWI-GEN study participants. In attendance were chiefs, elders and community members. On each occasion the NHRC team made presentations on the AWI-Gen study focusing mainly on the background and objectives of the study. Presentations also covered the behavioural and biological risk factors associated with cardiovascular disease (CVD), and the main findings of the study; putting emphasis on the aggregate findings for the KNDs, and general education on CVD preventive measures. The study team this time included a clinician, a native of the community and fluent in the local language to respond to issues related to clinical care for non-communicable diseases. The community members were given opportunity to ask questions, raise any concerns or make any comments regarding the project and adult health issues in general. Most of the questions and comments from the community members focused on the need for education on CVD preventive measures at the individual level and knowledge of the signs and symptoms and lifestyle factors associated with CVDs.

## Individual feedback

**Figure 2.**
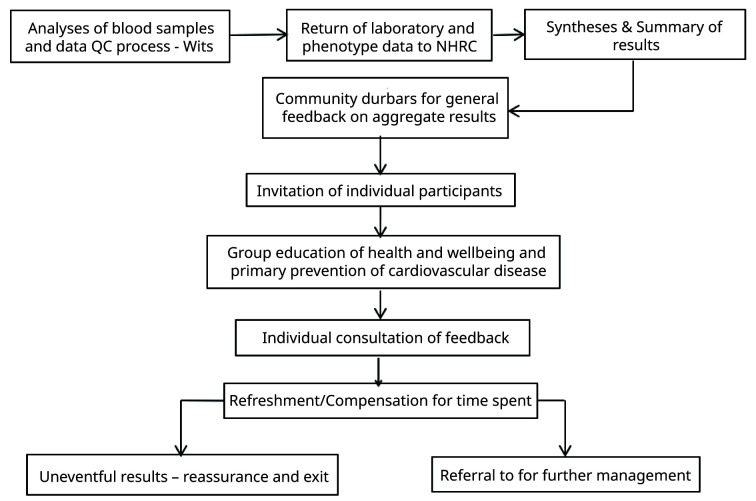
AWI-Gen community and individual feedback process. QC: quality control, NHRC: Navrongo Health Research Centre.

Following the general feedback to the study communities through community durbars, the next set of activities focused on giving back individual results to study participants. Prior to the commencement of this activity, a research assistant and a fieldworker were deployed to the study communities to locate the participants in the households and invite them to a common venue in the community on a scheduled date and time. Typical venues for the exercise were health centers, Community-based Health and Planning Services (CHPS) compounds ( Nyonator *et al.,* 2005) and schools. Such facilities were chosen to ensure some privacy for the clinician to discuss results with individuals. At each venue, participants were first given an overview of the AWI-Gen study and the main findings of the study similar to what was presented in the community durbars. Thereafter, individuals met with a clinician who interpreted their results to them in private and individually. Individuals with results outside the normal values were referred to appropriate health facilities by the clinician for further evaluation and care. All others were given education on preventive measures and general advice on how to stay healthy (See Figure 2).

All the 2016 individuals recruited in 2015 were targeted for invitation for individual feedback. A total of 1775 (88.0%) participants were met and provided individual feedback; 71 (3.5%) participants were reported dead, 64 (3.2%) had migrated out of the catchment area, while 62 (3.1%) were lost to follow up as they were not met during the household visit and did not turn up at the venues for their results. The high proportion who came for feedback indicated their continued interest in the study and its outcomes. In line with HDSS protocols verbal autopsies have been performed for the deceased and this will provide valuable input for the next phase of the AWI-Gen study in order to track health outcomes and causes of death.

## Challenges and lessons learned

The feedback and engagement activities presented several challenges. The team observed from their interaction with the community that participants were overly expectant with regard to feedback activities. This could be due to perceptions raised about the potential outcomes of the study during the community sensitization and engagement prior to participant recruitment. Some participants thought the test results would be given back to them within a short time frame and they had the impression that those needing treatment would have the cost borne by the NHRC. Nonetheless, the general community and the study participants appreciated the efforts of the study team in providing feedback on the study results. The following statement by a participant highlights the sentiments of participants:

“ *Now we know that VAST people don’t tell lies, if they say they will do something, they keep to their words. Who would ever think that three years after the work you will still follow to check on us?”* (quote from male AWI-Gen participant)

The timing for the feedback and engagement activities also presented some challenges. May to July form part of the rainy season in the Kassena-Nankana district and represents a period of intense farming activities. Apart from torrential rain disrupting the meetings occasionally, community members were also busy with farm work, and found the meetings disruptive of their activities. Another challenge was the migration of individuals out of the study area; this made it impossible to locate some participants. The team observed that organizing durbars at the chief palaces were less effective compared to a common venue outside of the palaces. This could probably be due to the perception by community members that meetings at the chief palaces are meant for elders and clan heads and not for ordinary community members, especially women.

In spite of these challenges the communities and participants were generally cooperative and punctual when invited for the feedback meetings. The warm reception of the chiefs and elders of the study communities was another major success. Despite the difficulties in locating some participants the HDSS made participant location generally efficient. Through this exercise community members within the study community were educated on lifestyle factors associated with CVDs and the basic preventive measures. Above all, the dissemination rekindled the trust the study communities have in the NHRC and this sets the stage for a successful recruitment exercise for the second phase of AWI-Gen. Some of the key recommendations drawn from these experiences include the following:

## Recommendations

- Adequate funding for CE activities should be budgeted for in research applications.
- There is the need to give immediate feedback of point-of care results in future projects.
- Future engagement activities should be carried out during the dry season when there are less agricultural or farming activities.
- Future dissemination of results and community engagement activities should be conducted in public spaces in the community where all members feel comfortable to attend and not at the chief palaces.
- During sensitization exercises prior to participant recruitment there is the need to avoid ‘exaggerated assurance’ of immediate feedback of all results and care should be taken to emphasize that health care may not be provided by research studies. This will avoid participants being overly expectant.
- Community engagement, informed consent and feedback of findings are closely related, particularly for community-based studies. When community engagement activities are conducted well, they can support the informed consent process and facilitate the feedback of research results to communities and research participants.

## Conclusion

The community engagement processes and experiences described in this paper highlight the importance of incorporating engagement as an integral part of the research process. Not only does this demonstrate respect to local communities, it also ensures that community concerns are adequately addressed to facilitate the ethical conduct of research. The feedback of results is important as it provides the platform for participants to get information on the tests performed on their samples and also provides an opportunity to receive further education on scientific research and sensitization on healthy living. With the limited scholarship on what and how to effectively return genomics results to participants, we encourage further empirical studies that will explore what communities really want when they ask for the return of research results and how research teams can respond to these expectations.

## Data availability

### Underlying data

No data are associated with this article.
