# Supplementary material for: Community engagement and feedback of results in the H3Africa AWI-Gen project: Experiences from the Navrongo Demographic and Health Surveillance site in Northern Ghana
Source: AAS Open Res. 2021 Mar 19;4:15. [Version 1] doi: 10.12688/aasopenres.13081.1 (PMC8063542; doi:10.12688/aasopenres.13081.1)
Supplement: Peer review history

# Peer review: Reviewer response for version 1

**DOI:** 10.21956/aasopenres.14177.r28504

**Published:** 2021-04-13

**Authors:** Rosemary Musesengwa

**Affiliations:** 1 Department of Psychiatry, Wellcome Centre for Ethics and Humanities, University of Oxford, Oxford, UK

This is a very well written simple letter highlighting some key issues in Community Engagement and Feedback of Results.

**Abstract:**

- I would recommend the team acknowledges that there is a considerable amount of literature and guidance for community engagement now available for research teams to use but the issue is that more is required since CE tends to be highly contextual and researchers will benefit from many different perspectives and experiences, including this one. 

**Introduction:**

- The team could add some information on the literature or guidance documents that guided their CE approach. 

**CE Processes**

- The processes are well outlined and have very clear diagrammatic representations of the process, easy for researchers to follow.

**Limitations:**

- Whilst it is a letter, I still feel the team could have highlighted the shortcomings of their work as well. It seems to me that the bulk of their challenges were bought about by a seemingly lack of thorough formative research before they started the study. Unless I misinterpreted or missed it somewhere, I feel this should have been highlighted as one of the key lessons. It needs to be stated in that there is need for prior formative work to understand community expectations at the beginning of a study in order to address them as they progress. 

Does the article adequately reference differing views and opinions?

No

Are all factual statements correct, and are statements and arguments made adequately supported by citations?

Partly

Is the Open Letter written in accessible language?

Yes

Where applicable, are recommendations and next steps explained clearly for others to follow?

Yes

Is the rationale for the Open Letter provided in sufficient detail?

Partly

Reviewer Expertise:

Community Engagement

I confirm that I have read this submission and believe that I have an appropriate level of expertise to confirm that it is of an acceptable scientific standard, however I have significant reservations, as outlined above.

# Peer review: Reviewer response for version 1

**DOI:** 10.21956/aasopenres.14177.r28503

**Published:** 2021-04-09

**Authors:** Noni Mumba

**Affiliations:** 1 Health Sysytems and Research Ethics, Center for Geographical Medicine, KEMRI-Wellcome Trust Research Programme, Kilifi, Kenya

I enjoyed reading the publication. It is well written and clear. It is interesting to me to note that community level engagement has a lot of similarities across different African contexts; for instance, similar community entry systems.

 The authors have arranged their paper well, outlining the different stages they went through from the start to the end.

**Introduction:**

 Please include a brief outline of the study itself, especially covering study procedures. This is an important part of engagement, and it would be interesting to see specific examples of how study procedures were explained during community durbars. For example, what terms were used to explain genetic testing?

**Community Entry:**

 Was there any role of local health gatekeepers? Community engagement usually broadly includes local health gatekeepers such as local public health officers; however the paper only outlines the grassroots leaders.

**Study participants:**

 I do not understand what *'30 trios means*'

**Figure 1:**

 This is outlining more of study procedures than engagement process.

**CE Processes:**

 It would be good to explain further who from the study team participated in engagement activities. Often, we encourage the PIs to participate, but recognize that they are sometimes very busy. 

**Recommendations:**

 In my view, the authors should try and make these as broad as possible. They seem to be written specifically to suit the context they are in. They can be rephrased to read like recommendations that can apply in any context (for example bullet 3 can read: "As engagement plans are made, teams must take into account climatic/weather conditions and accompanying social activities like planting)...".

 I did not understand the term ' *exeggerated assurance*'.

Does the article adequately reference differing views and opinions?

Partly

Are all factual statements correct, and are statements and arguments made adequately supported by citations?

Yes

Is the Open Letter written in accessible language?

Yes

Where applicable, are recommendations and next steps explained clearly for others to follow?

Partly

Is the rationale for the Open Letter provided in sufficient detail?

Yes

Reviewer Expertise:

Community, Public and Policy engagement; Ethics

I confirm that I have read this submission and believe that I have an appropriate level of expertise to confirm that it is of an acceptable scientific standard.

# Peer review: Reviewer response for version 1

**DOI:** 10.21956/aasopenres.14177.r28502

**Published:** 2021-03-29

**Authors:** Erisa Mwaka

**Affiliations:** 1 Department of Anatomy, School of Biomedical Sciences, Makerere University College of Health Sciences, Kampala, Uganda

I would like to commend the authors for the manuscript. Community engagement is very key to responsible conduct of research. However, the nature of the findings that were returned to participants and their communities is not clear. It is also not clear whether genetic results were returned. It is important for the authors to indicate whether genetic results were returned because the return of such results involves a lot.

 Below are the detailed comments:

- More information should be given on the AWI-Gen study in the introduction. The information should include the overarching objective and a brief paragraph on study procedures so that the reader from the outset understands what that study was all about. This is important because later on in the manuscript, the authors introduce the concept of return of results. However, it is not clear whether any genetic results were obtained.
- In the introduction "30 trios" appear: what is 30 trios?
- In the introduction the authors indicate that this was a multi-national project involving Kenya, South Africa, Ghana and Burkina Faso. Which country are Kassena-Nankana districts? This should also be clearly indicated in the introduction so that it is clear from the outset.
- More detail should be provided on the selection criteria of the homes visited, this is unclear.
- It is not clear which particular results were fed-back at the community durbars. The authors state that emphasis was placed on aggregate findings; were these aggregate genetic findings?
- Individual results: Were these results of laboratory tests or Genetic results?
- If genetic, were the results validated? Were genetic counseling services available or they were not necessary?
- Did all participants gracefully receive their results or were there some ethical and social issues that arose from receiving these results by the participants?
- Were participants re-consented to receive the results or the original consent for participation in the AWIGen study included a section on how results would be handled?

Does the article adequately reference differing views and opinions?

Partly

Are all factual statements correct, and are statements and arguments made adequately supported by citations?

Yes

Is the Open Letter written in accessible language?

Yes

Where applicable, are recommendations and next steps explained clearly for others to follow?

Yes

Is the rationale for the Open Letter provided in sufficient detail?

Yes

Reviewer Expertise:

Ethical, legal and societal implications of biobank and genetic/genomic research.

I confirm that I have read this submission and believe that I have an appropriate level of expertise to confirm that it is of an acceptable scientific standard, however I have significant reservations, as outlined above.
